# Activity-Dependent Bidirectional Regulation of GAD Expression in a Homeostatic Fashion Is Mediated by BDNF-Dependent and Independent Pathways

**DOI:** 10.1371/journal.pone.0134296

**Published:** 2015-08-04

**Authors:** Yoko Hanno-Iijima, Masami Tanaka, Takatoshi Iijima

**Affiliations:** 1 Tokai University Institute of Innovative Science and Technology, Medical Division, Kanagawa, Japan; 2 School of Medicine, Tokai University, Kanagawa, Japan; University of Louisville, UNITED STATES

## Abstract

Homeostatic synaptic plasticity, or synaptic scaling, is a mechanism that tunes neuronal transmission to compensate for prolonged, excessive changes in neuronal activity. Both excitatory and inhibitory neurons undergo homeostatic changes based on synaptic transmission strength, which could effectively contribute to a fine-tuning of circuit activity. However, gene regulation that underlies homeostatic synaptic plasticity in GABAergic (GABA, gamma aminobutyric) neurons is still poorly understood. The present study demonstrated activity-dependent dynamic scaling in which NMDA-R (*N*-methyl-D-aspartic acid receptor) activity regulated the expression of GABA synthetic enzymes: glutamic acid decarboxylase 65 and 67 (GAD65 and GAD67). Results revealed that activity-regulated BDNF (brain-derived neurotrophic factor) release is necessary, but not sufficient, for activity-dependent up-scaling of these GAD isoforms. Bidirectional forms of activity-dependent GAD expression require both BDNF-dependent and BDNF-independent pathways, both triggered by NMDA-R activity. Additional results indicated that these two GAD genes differ in their responsiveness to chronic changes in neuronal activity, which could be partially caused by differential dependence on BDNF. In parallel to activity-dependent bidirectional scaling in GAD expression, the present study further observed that a chronic change in neuronal activity leads to an alteration in neurotransmitter release from GABAergic neurons in a homeostatic, bidirectional fashion. Therefore, the differential expression of GAD65 and 67 during prolonged changes in neuronal activity may be implicated in some aspects of bidirectional homeostatic plasticity within mature GABAergic presynapses.

## Introduction

Homeostatic synaptic plasticity tunes synaptic strength to compensate for prolonged, excessive changes in neuronal activity [1]. This plasticity is also thought to play an important role in the maintenance of proper network activity. Several studies have revealed the precise mechanisms underlying activity-dependent changes in strength at glutamatergic synapses. For one, activity-dependent scaling of excitatory synapses depends on postsynaptic activity [2]. Furthermore, prolonged changes in neuronal activity lead to alterations in the number of synaptic α-amino-3-hydroxy-5-methyl-4-isoxazole propionic acid receptors (AMPA-Rs) through protein synthesis-dependent AMPA-R trafficking; this, in turn tunes excitatory synaptic transmission [3]. However, not only is excitatory synaptic strength tuned but also inhibitory synaptic strength [4] [5]. The γ-aminobutyric acid (GABA)-type inhibitory neuron regulates synaptic integration, the probability of action potential generation, and the timing of action potential generation. Impaired inhibitory functioning is one of the underlying contributory causes of neuropsychiatric disorders, as well as neuropathies characterized by brain ischemia and neuronal hyper excitability syndromes such as seizure and epilepsy [6]. Therefore, it is expected that homeostatic control of inhibitory neurons will be revealed as very important for overall network activity maintenance within neuropathological conditions. However, the gene expression changes underlying homeostatic changes at GABAergic inhibitory synapses remain unclear.

One of the key factors for gene regulation during homeostatic scaling within inhibitory synapses is brain-derived neurotrophic factor (BDNF) [7] [8], which is predominantly expressed in excitatory neurons [9] [10] [11] [12]. Neuronal activity promotes BDNF expression [13] [14] [15], and a GABA-synthetic enzyme—glutamic acid decarboxylase (GAD)—is one of the major factors upregulated by long-term BDNF treatment via MAP kinase [16] [17]. GAD transcription is induced by BDNF-TrkB signaling in a RAS-ERK-CREB dependent manner [17]. Therefore, the present study’s main hypothesis was that any Ca^2+^-dependent dynamic GAD up-regulation is mediated by NMDA-induced BDNF that is produced and released from surrounding excitatory neurons. In fact, there is increasing evidence for a relationship between homeostatic scaling at GABAergic inhibitory synapses and BDNF-induced GAD expression. However, it remains unclear whether BDNF release is sufficient for activity-dependent GAD expression and the homeostatic functions of GABAergic inhibitory neurons. In this regard, it is relevant that GAD has two isoforms—GAD65 and GAD67—encoded by two separate genes, *Gad2* and *Gad1*, respectively [18]; these isoforms also have different subcellular localizations and functions [18] [19] [20]. Therefore, the present study sought whether there are any differences in activity-dependent gene regulation and related neuronal functions between these two GAD isoforms.

The present study revealed that activity-dependent dynamic regulation of GAD65 and GAD67 is differentially controlled by Ca^2+^-dependent mechanisms triggered by *N*-methyl-D-aspartic acid receptors (NMDA-Rs). Results confirmed that activity-regulated BDNF could be a key factor in this process. However, BDNF is not sufficient for full bidirectional regulation, suggesting that some Ca^2+^-dependent, and BDNF-independent, pathway is also necessary for the activity-dependent control of GAD expression. It was also observed that responsiveness to neuronal activity is distinctly different between these two GAD isoforms, which could be due to a differential dependence on the BDNF signal. Overall, these findings imply that the interplay of activity-dependent expressions between these two GAD isoforms may be a plausible mechanism underlying dynamic homeostatic plasticity at GABAergic presynapses.

## Materials and Methods

The present study assessed the effect of various pharmacological inhibitors and blockers of NMDA-dependent and/or BDNF-mediated signaling in cultured cortical neurons in the presence of chronically increased or decreased activity due to bicuculline or tetrodotoxin (TTX), respectively. Gene expression changes were evaluated at both RNA and protein levels. Protein expression was evaluated by both western blot and immunostaining. Synaptic functioning was measured by depolarization-evoked release of GABA and glutamate into the culture medium. Cultured neurons were treated with TTX or bicuculline by adding the drugs to the culture medium and incubating for 1–4 days at 37°C. Blocking agents, such as the MAP kinase inhibitor (U0126) and the selective CaM kinase inhibitor (KN93), were added with the bicuculline or TTX to further elucidate the signaling pathways.

### Neuronal cell culture

Cortical neuron cultures were prepared from ICR mouse pups on embryonic day 15 by tissue dissociation with 0.05% trypsin (Sigma) in the presence of DNase I (Roche Applied Science) for 10 min at 37°C. After cell dissociation, trypsin was inactivated with soybean trypsin inhibitor (Sigma). Cells were then plated into 24-well dishes (0.5–1.0 × 10^5^/cm^2^) and maintained for 15 days in Neurobasal Medium (Invitrogen) containing 2% B27 supplement, 2 mM Glutamax, and penicillin/streptomycin (Invitrogen). All procedures related to the care and treatment of animals was carried out in strict accordance with recommendations from the Guide for the Care and Use of Laboratory Animals of Tokai University. All mice were maintained under specific pathogen-free conditions at the Laboratory Animal Center, Tokai University. The Institutional Animal Care and Use Committee of Tokai University (permit number: 141018) approved the protocol. All surgery was performed under sodium pentobarbital anesthesia, and all efforts were made to minimize suffering.

### Measurement of depolarization-induced neurotransmitter release from the cultured neurons

Before stimulation, the treatment drugs were washed out with fresh Neurobasal Medium, and then the evoking stimulus (the depolarization agent 4-AP) was applied for 3 min. GABA and glutamate release evoked from TTX- or bicuculline-treated neurons were measured using high performance liquid chromatography (HPLC; Shimadzu Co., Kyoto, Japan).

### Drugs

4-AP was purchased from Sigma. AP5, BDNF, bicuculline, K252a, KN93, NBQX, TTX, and U0126 were purchased from TOCRIS. BDNF was from Peprotech.

### Protein analyses

For quantitative western blotting, 15 μg of total protein were separated by polyacrylamide gel electrophoresis and transferred onto nitrocellulose. For visualization, horseradish peroxidase (HRP)-conjugated secondary antibody and enhanced chemoluminescent detection (ECL; Pierce) were used. Signals were acquired using an image analyzer (LAS-3000; Fujifilm) and quantified with ImageJ Software (NIH). Signal intensities were then normalized to the internal control (GAPDH).

### RNA analyses

Total RNA was isolated using a Trizol reagent (Invitrogen), followed by removal of contaminating DNA with Turbo DNA-free (RNase-free DNase; Ambion). One microgram of total RNA was reverse transcribed using random hexamers and ImPromII (Promega). Quantitative PCR was performed on a StepOnePlus qPCR system (Applied Biosystems). The custom primer sets (see Table 1) were used with SYBR Green Master Mix (Applied Biosystems) and the comparative C^T^ method. The mRNA levels were normalized to that of *Gapdh* mRNA.

**Table 1:** Oligonucleotide sequences of the qPCR primer sets.

**primer**		**Sequence (5'-3')**
Vglut1	forward	5'- TCA AGG CTC GCC TAA CCA ATT C -3
	reverse	5'- TTT CCC TCA GAA ACG CTG GTG -3'
Vgat	forward	5'- TCC TGG TCA TCG CTT ACT GTC TC-3’
	reverse	5'- CGT CGA TGT AGA ACT TCA CCT TCT C-3’
Gad65	forward	5'- CCT GGT TAG AGA GGA GGG ACT GA -3’
	reverse	5'- CAT AGT GCT TAT CTT GCT GAA AGA GGT A-3’
Gad67	forward	5'- GCG GGA GCG GAT CCT AAT A-3’
	reverse	5'- TGG TGC ATC CAT GGG CTA C-3'
Gapdh	forward	5'- TGT TCC AGT ATG ACT CCA CTC ACG -3'
	reverse	5'- AGT AGA CTC CAC GAC ATA CTC AGC -3’

### Immunocytostaining, image acquisition, and analyses

Cultured neurons were fixed with ice-cold fixative (4% paraformaldehyde in 100 mM phosphate buffer, pH = 7.4) for 20 min and then permeabilized with PBS containing 0.1% TritonX-100 for 15 min at room temperature. Cells were then incubated with blocking solution (5% normal donkey serum in PBS) for at least 30 min at room temperature and were then incubated with the primary antibodies for 24 h at 4°C. For visualization, appropriate secondary antibodies conjugated to Alexa 546 or 488 (goat, 1:2,000) (Invitrogen) were used. Nuclear staining was performed with DAPI (DAKO). Confocal images were captured on a Zeiss LSM5 confocal system (Zeiss) and were assembled using Adobe Photoshop and Illustrator Software. Images were analyzed using MetaMorph Software (Molecular Dynamics). A single threshold was set for each staining condition to capture puncta that were clearly distinguishable and to minimize counting merged structures. For quantification of GAD67- and vGluT1-positive areas, total positive puncta area was measured in randomly selected fields from each group.

### Antibodies

The rabbit anti-BDNF antibody was a kind gift from Dr. Barde. The following commercially available antibodies were used: mouse anti-GAD (MBL), mouse anti-GAD65 (Developmental Studies Hybridoma Bank), mouse anti-GAD67 (Millipore), rabbit or guinea pig anti-vesicular glutamate transporter 1 (vGluT1), rabbit anti-vesicular GABA transporter (vGAT), mouse anti-NR1 (Synaptic Systems), anti-NR2A (Upstate Biotechnology), mouse anti-NR2B (Neuromab), rabbit anti-GAPDH (EnoGene Biotech Co.), mouse anti-actin (Sigma), anti-NeuN (Millipore) and mouse anti-MAP2 (Sigma).

Secondary antibodies with minimal interspecies cross-reactivity conjugated to cyanine and Alexa 633, 546, or 488 (dyes obtained from Jackson ImmunoResearch and Invitrogen) were used for visualization. The secondary HRP-conjugated anti-mouse and anti-rabbit IgG used for western blotting were purchased from Jackson.

### Statistical analyses

All statistical analyses were performed using Prism 5 (GraphPad Software). Pairwise comparisons were performed using Student's *t*-test. For multiple comparisons, analysis of variance (ANOVA) followed by a Bonferroni or Dunnett test was used. Data are represented as the mean ± SEM. Significance is indicated as follows: ***, *p* < .001; **, *p* < .01; *, *p* < .05; n.s., no significant difference.

## Results

### Different forms of activity-dependent bidirectional expression between GAD65 and GAD67

**Fig 1:**
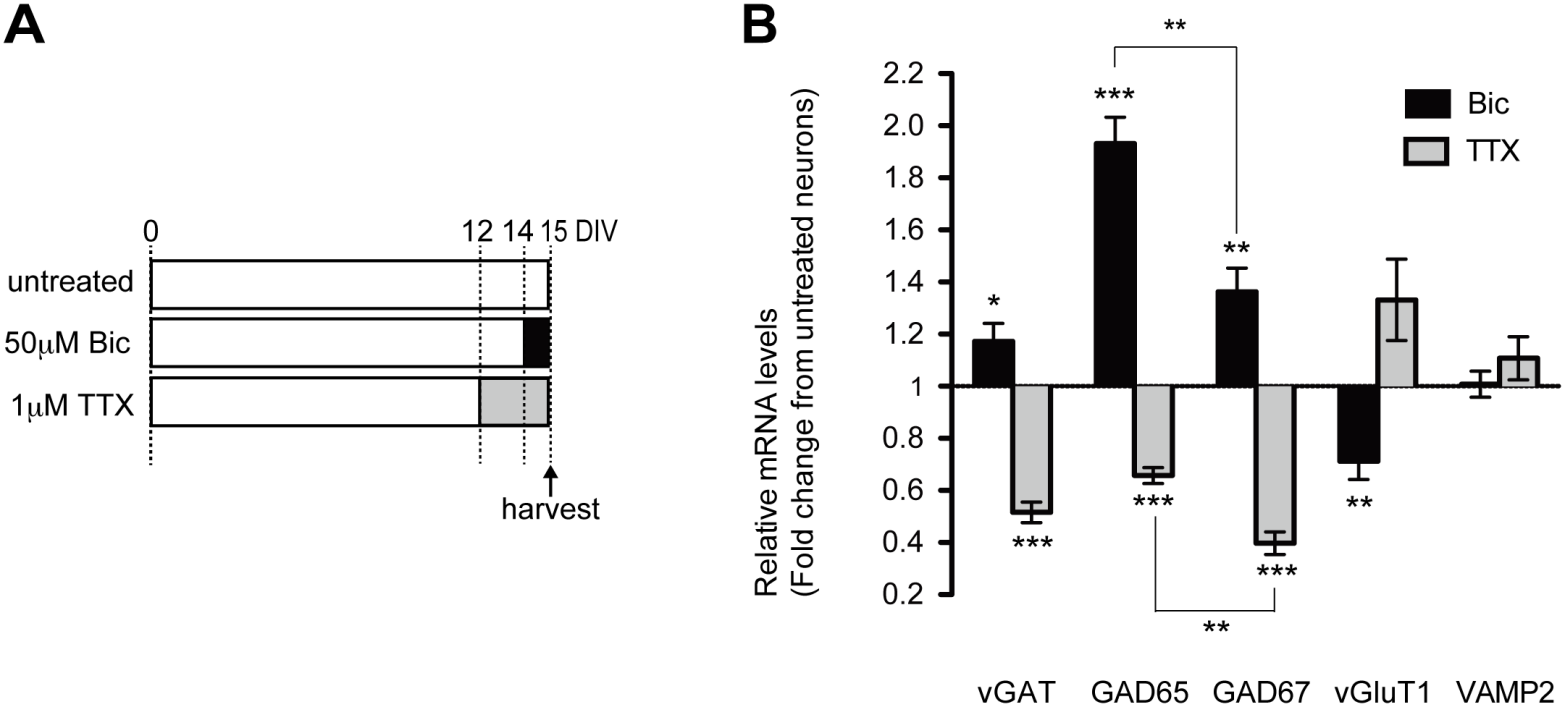
Different bidirectional forms of activity-dependent gene regulation governing GAD expression. (A) Cultured cortical neurons were treated with 50 μM bicuculline on DIV14 or with 1 μM TTX on DIV12 and were harvested on DIV15. (B) Shows reciprocal effects of bicuculline and TTX on relative expression levels of mRNAs encoding GABAergic and glutamatergic presynaptic proteins in cultured cortical neurons treated as shown in (A). GAD67: F(2, 14) = 106.5 (*p* < .0001); GAD65: F (2, 14) = 62.36 (*p* < .0001); vGluT1: F(2, 14) = 11.52 (*p* = 0.001); VAMP2: F(2, 14) = 1.26 (no significant difference, *p* = 0.31), one-way ANOVA. (*n* = 5–6 in each group). GAD: glutamic acid decarboxylase; DIV: days in vitro; TTX: tetrodotoxin.

To examine GAD gene regulation details during chronic neuronal activity changes, we first checked the level of GAD mRNAs in cultured cortical neurons treated with bicuculline and tetrodotoxin (TTX). This was done along with vesicular presynaptic protein levels regulated by neuronal activity, as reported previously [21] (Fig 1A). Our qPCR analyses revealed that mRNA levels of the two GAD isoforms were significantly increased in bicuculline-treated cultures compared to untreated ones (GAD65 1.9 ± 0.1 fold, *p* < .001; GAD67, 1.36 ± 0.09 fold, *p* < .01) and significantly decreased in TTX-treated cultures (GAD65 0.66 ± 0.03 fold, *p* < .001; GAD67, 0.40 ± 0.04 fold, *p* < .001) (Fig 1B). Thus, both GADs showed a bidirectional form of activity-dependent change. In parallel, we also observed significant activity-dependent change in the mRNA levels of vesicular GABA transporter (vGAT) and vesicular glutamate transporter 1 (vGluT1), as reported previously [21]. However, this alteration was partial or smaller than that of the two GAD isoforms. Synaptobrevin (VAMP2) mRNA level was not significantly altered.

Additionally, we noticed that bidirectional responsiveness to change in activity levels was distinctly different between the two GAD genes (Fig 1B). Increased activity due to bicuculline preferentially up-regulated GAD65 (*p* < .01), whereas activity deprivation due to TTX preferentially down-regulated GAD67 (*p* < .01). These results imply that activity-dependent bidirectional changes in the expression of the two GAD genes are regulated by different mechanisms.

### Increased activity up-regulates GAD expression through NMDA-R activation

**Fig 2:**
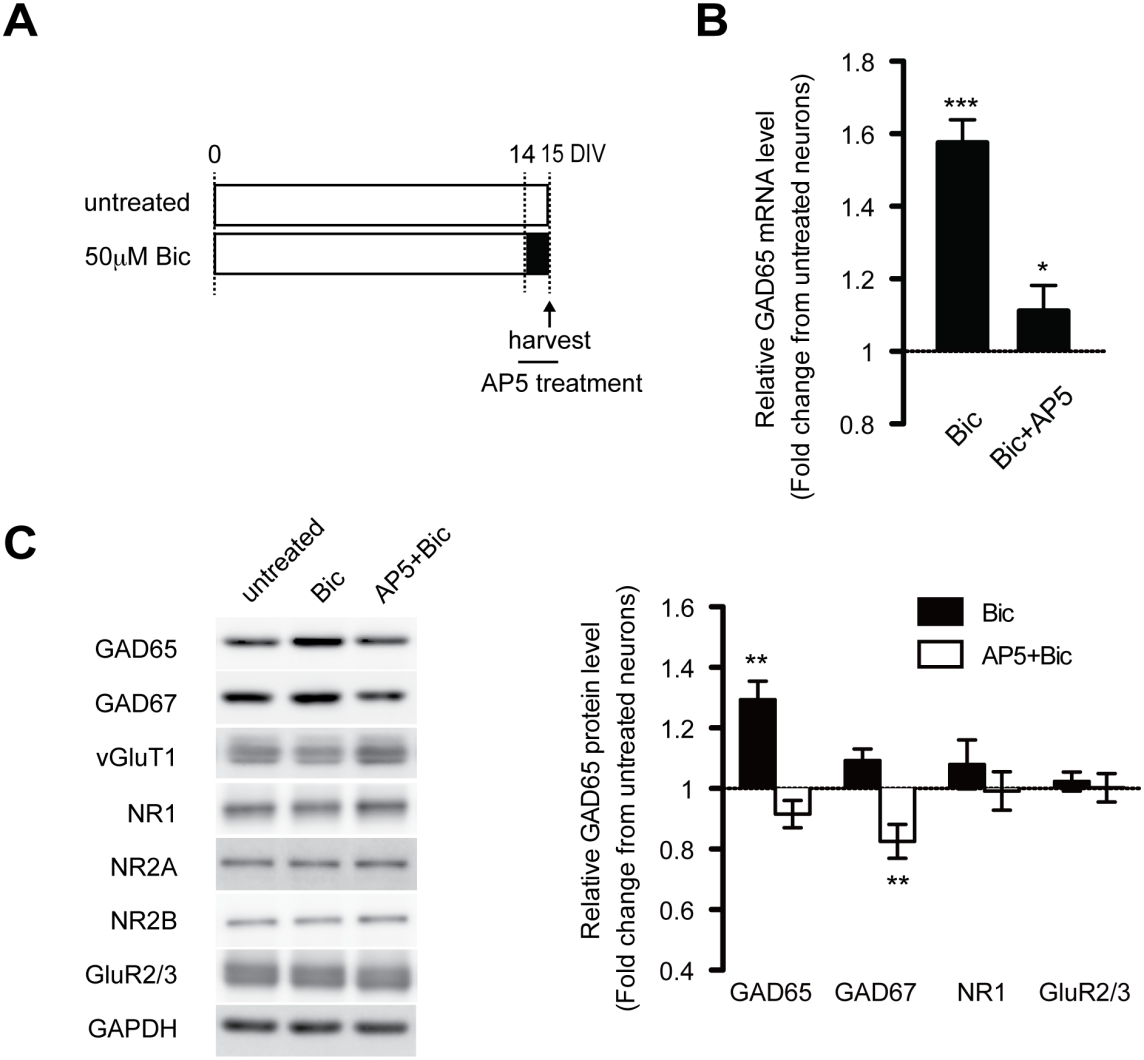
Chronically increased activity enhances GAD expression via a Ca^2+^-dependent mechanism triggered by NMDA-R activation. (A) Cultured cortical neurons were treated with 50 μM bicuculline in the presence or absence of AP5 (200 μM) on DIV14 for the final day in the culture and were harvested on DIV15. (B) Shows relative mRNA expression levels of GAD65 in cultured cortical neurons treated as shown in (A). F(2, 46) = 43.72 (*p* < .0001), one-way ANOVA. (*n* = 3–6 in each group). (C) Relative protein levels of GAD65, GAD67, NR1, and GluR2/3 measured by immunoblotting. Bicuculline induced a significant increase in GAD65 protein that was completely blocked by AP5. GAD67: F(2, 25) = 14.09; GAD65: F (2, 23) = 21.78 (*p* < .0001), one-way ANOVA. (*n* = 3–5 in each group). Bic: bicuculline.

We next checked the NMDA-R–mediated Ca^2+^ dependence of GAD expression. Increased expression of GAD65 with bicuculline treatment was dramatically reduced by the application of AP5, a selective blocker of Ca^2+^-permeable ion channels and NMDA-Rs (Fig 2A and 2B). This confirmed that GAD expression is actually dependent on NMDA-Rs. The same results were confirmed at the protein level. While bicuculline treatment strongly increased GAD65 protein by immunoblot analysis, this effect was completely blocked by AP5 (Fig 2C). However, while GAD67 definitely had a tendency to be up regulated, we did not observe a significant increase at the protein level (Fig 2C). In contrast, neither bicuculline nor AP5 significantly affected the protein level of any NMDA-R subunit tested (i.e., NR1, NR2A, and NR2B) (Fig 2C). Additionally, we showed that bicuculline-induced increases in GAD65 expression were nearly completely blocked with the transcription inhibitor actinomycin D (S1 Fig). These data reveal that dynamic changes in GAD expression are largely regulated through NMDA-R activity on the transcriptional level.

### BDNF-TrkB signaling is involved in activity-dependent up-regulation of GAD65 expression upon increased activity

**Fig 3:**
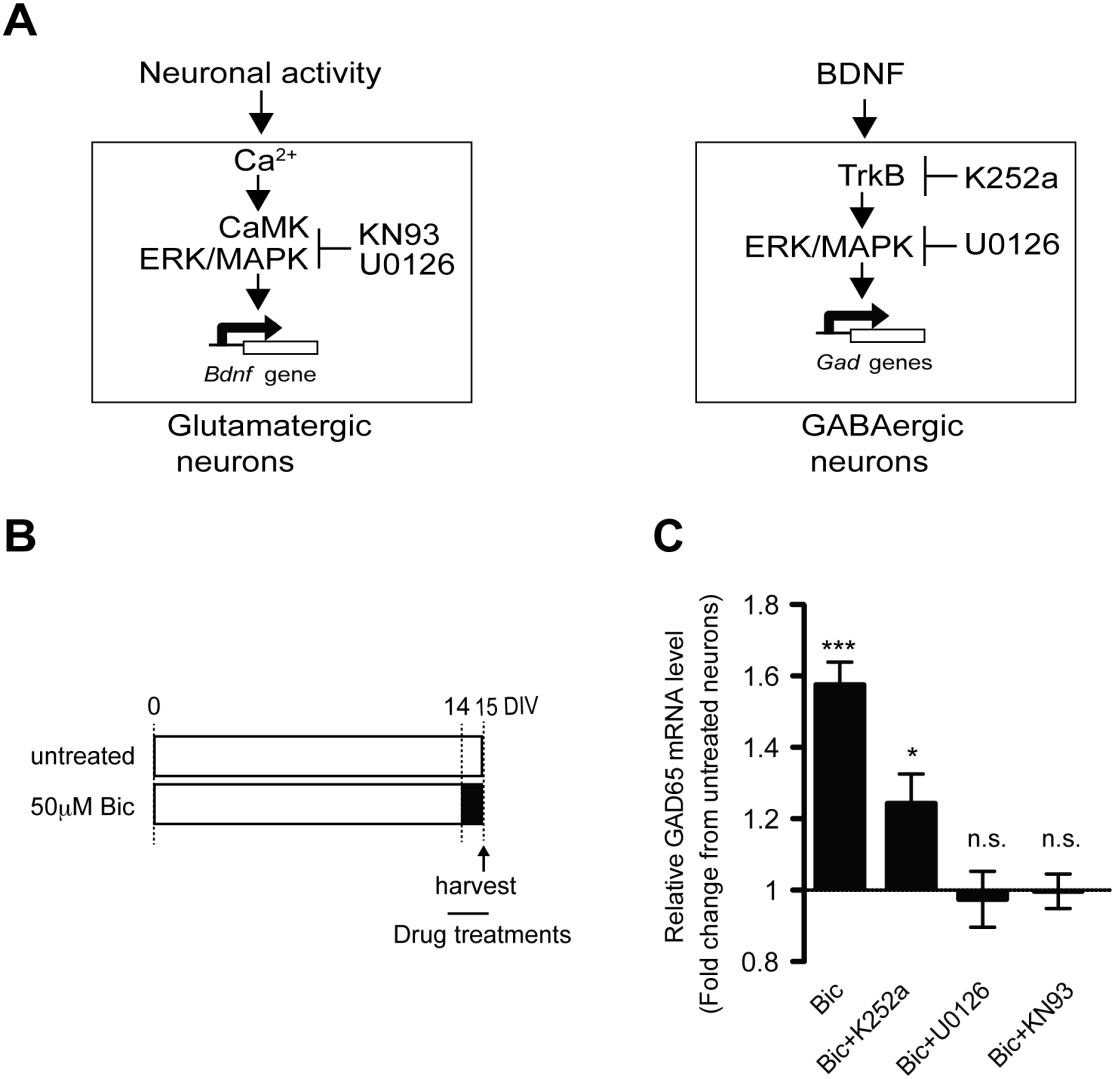
Chronically increased activity enhances expression of GAD65 via an NMDAR-dependent mechanism through BDNF-TrkB signaling. (A) Predicted molecular pathways of activity-dependent GAD expression mediated through BDNF in a non-cell-autonomous manner, and the related pharmacological inhibitors of NMDA-R-dependent Ca^2+^ and BDNF signals. (B) K252a (100 nM), U0126 (10 μM), AP5 (200 μM) or KN93 (5 μM) were co-applied to cultured cortical neurons on DIV14 with 50 μM bicuculline. The neurons were harvested 24 hours after drug treatment. (C) Relative GAD65 mRNA expression levels after treatment with bicuculline with and without inhibitors as shown in (B). F(4, 62) = 26.88 (*p* < .0001), one-way ANOVA (*n* = 4–10 in each group). BDNF: brain-derived neurotrophic factor.

To test our initial hypothesis that NMDA-R activity-dependent GAD up-regulation is mediated by BDNF, we assessed the effect of various inhibitors or blockers of NMDA-dependent pathways and/or downstream pathways of BDNF in cultured cortical neurons upon increased activity with bicuculline (Fig 3A and 3B). We initially confirmed that, in parallel to up-regulation of GAD expression, bicuculline treatment promoted BDNF expression (S2A Fig). The increase in BDNF expression was blocked by the MAP kinase inhibitor, U0126, and the selective CaM kinase inhibitor, KN93 (S1A Fig). Similarly, bicuculline-induced up-regulation of GAD65 was completely blocked by U0126 and KN93 (Fig 3D), revealing that GAD expression is highly related to the expression level of BDNF. Notably, bicuculline-induced up-regulation of GAD65 was also significantly attenuated by K252a, a selective inhibitor of the tyrosine protein kinase activity of the Trk family members, including BDNF receptors, TrkB (Fig 3D). However, K252a did not completely block bicuculline-induced up-regulation. This partial effect could not be due to low levels of BDNF expression because a significant amount of BDNF was produced with bicuculline in both the presence and absence of K252a. This confirmed that K252a itself does not disrupt activity-dependent BDNF expression at either the mRNA or the protein level (S2A and S2B Fig). Thus, these results suggest that GAD expression was enhanced by BDNF-TrkB signaling in the presence of persistently increased activity, but BDNF might not be a sufficient mediator of full activity-dependent GAD65 expression.

### Increased activity by bicuculline treatment enhances released BDNF and differentially regulates GAD expression with varied responsiveness to BDNF signaling between GAD65 and GAD67

**Fig 4:**
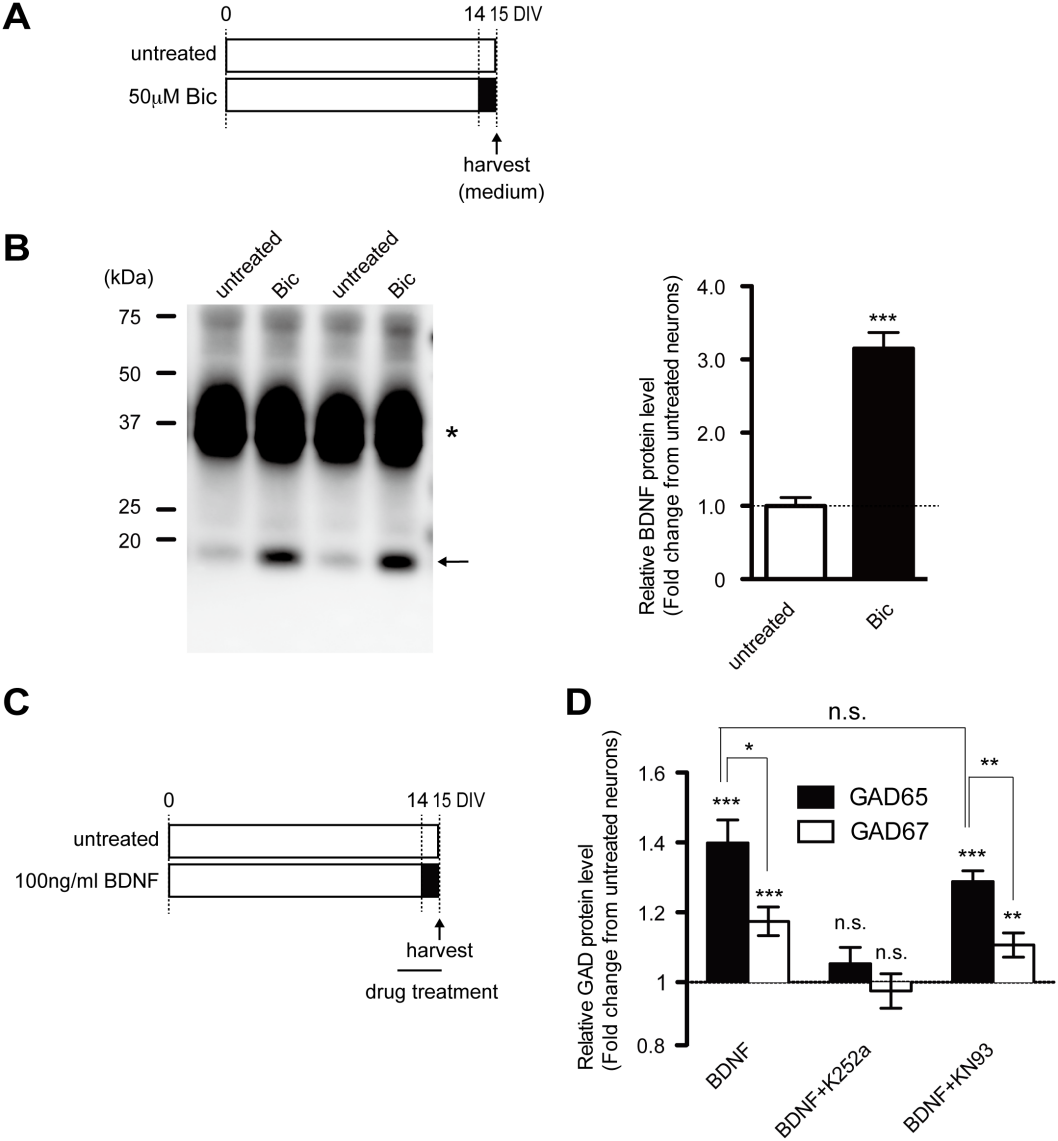
Released BDNF differentially accelerates expression of GADs under conditions of increased activity. (A) Cultured cortical neurons were treated with 50 μM bicuculline on DIV14 for the final day in the culture, and the conditioned medium was harvested on DIV15. (B) Level of released BDNF from cultured cortical neurons after treatment with bicuculine as shown in (A). *(n* = 6) Arrow shows mature BDNF. Note that the asterisk is an unknown, unspecific band (not pro-BDNF) because a previous study used the same antibody that was detected in BDNF knockout mice. [22] (C) Recombinant human BDNF (100 ng/mL) is applied to cultured cortical neurons for 24 hours with or without inhibitors. (D) Relative GAD protein levels after treatment with BDNF. The increased expression of GAD65 and GAD67 is inhibited by K252a but not by KN93. Note that BDNF preferentially enhances GAD65 expression over that of GAD67. GAD67: F(3, 38) = 19.35 (*p* < .0001); GAD65: F(3, 25) = 21.78 (*p* < .0012), one-way ANOVA. *(n* = 6–10 in each group).

Next, we addressed whether “activity-regulated” BDNF contributes to GAD expression. In this case, activity-dependent increases in both BDNF mRNA and protein level would result in more BDNF release, which subsequently would activate downstream TrkB signaling. To this end, we checked BDNF levels released from bicuculline-treated cultured neurons. The actual amount of BDNF released into the cultured medium was dramatically increased in the bicuculline-treated culture (Fig 4A and 4B), implying that GAD expression could be promoted through activity-regulated BDNF-TrkB signaling.

Fig 1 illustrates that responsiveness to bicuculline-induced chronic activity is different between the two GAD genes. Therefore, we applied recombinant BDNF to cultured cortical neurons for 24 hours (Fig 4C) and then compared responsiveness between the two genes. BDNF application led to increases in the protein levels of both GAD isoforms (GAD65, 1.40 ± 0.07 fold, *p* < .001; GAD67, 1.17 ± 0.04 fold, *p* < .001) (Fig 4D). This increase was significantly blocked by K252a, but not KN93, consistent with a previous finding showing that BDNF-dependent synthesis of GAD65 is selectively mediated via the TrkB-ERK/MAPK pathway [16] [17]. Notably, we found that this BDNF-induced increase in the GAD67 protein was markedly lower than that of GAD65 (GAD65 vs. GAD67, *p* < .05) (Fig 4D), indicating that BDNF differentially regulates activity-dependent gene expression of the two GAD isoforms. Therefore, the different expression levels of the two GAD isoforms in a bicuculline-treated culture shown in Fig 1 might be explained by this varied dependency on BDNF.

### Activity deprivation reduces basal expression of GADs through the inhibition of NMDA-R-dependent and BDNF-independent mechanisms

**Fig 5:**
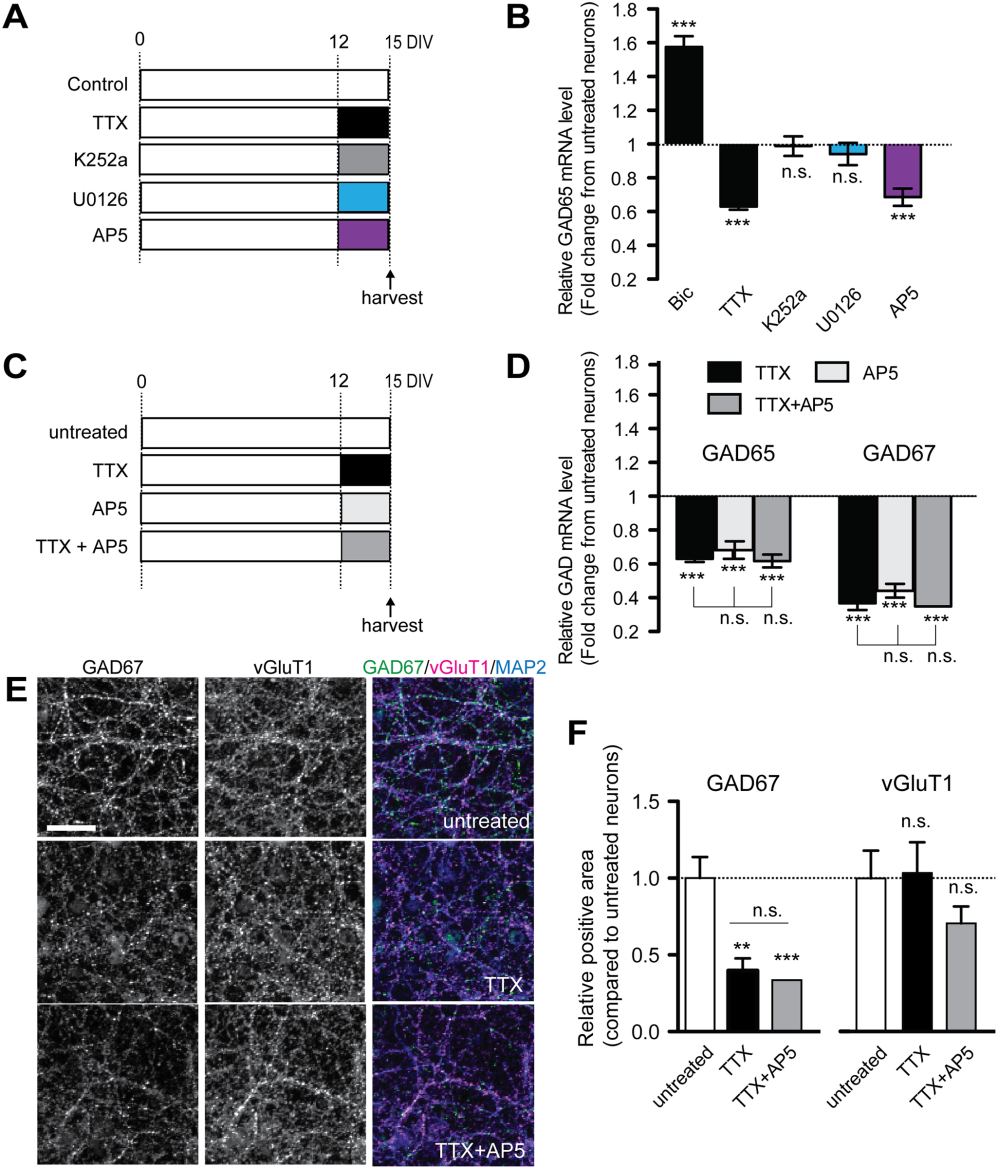
Activity deprivation reduces GAD expression with NMDA-R-dependent and BDNF-independent regulation. (A) Cultured cortical neurons are treated with bicuculline (50 μM), TTX (1 μM), K252a (100 nM), U0126 (10 μM), or AP5 (200 μM) for the last 3 days in a culture and are harvested on DIV15. (B) Relative GAD65 mRNA expression levels in neurons treated as shown in (A). The basal expression level of GAD65 is selectively reduced by inactivity caused by TTX or AP5 but not by the blockade of BDNF-TrkB signaling with K252a or U0126. F(5, 56) = 40.42 (*p* < .0001) one-way ANOVA. (*n* = 4–6 in each group). (C) Cultured cortical neurons are treated with TTX (1 μM), d,l-AP5 (200 μM), or co-treated with TTX and d,l-AP5 (200 μM) on DIV12 and are harvested on DIV15. (D) Relative GAD mRNA expression levels in cultured cortical neurons treated as shown in (C). The inhibitory effect of long-term TTX on GAD expression is completely occluded by AP5. GAD67 shows significantly decreased expression in TTX-treated neurons compared with GAD65. GAD67: F(2, 9) = 206.5; GAD65: F (2, 9) = 79.05 (*p* < .0001), one-way ANOVA. (*n* = 4 in each group). (E) Representative images of endogenous GAD67 and vGluT1 protein immunocytochemistry in TTX- and TTX plus AP5-treated cultures. Cultured cortical neurons are triple-stained with anti-GAD67, anti-vGluT1, and MAP2 antibodies. (F) Intensities of GAD67 and vGluT1 immunoreactivity measured in MAP2-positive dendritic areas. Co-application of TTX and AP5 does not have an additive effect on reducing GAD67 immunoreactivity. Twelve confocal images in each condition were analyzed from 3 independent experiments. GAD67: F(2, 23) = 14.40 (*p* < .0001); vGluT1: F(2, 33) = 1.152 (no significant difference, *p* = 0.3), one-way ANOVA. Scale bar = 50 μm.

We next focused on elucidating the mechanism underlying reduced expression of GADs upon activity deprivation with TTX. To this end, we assessed the single effects of various pharmacological inhibitors of NMDA-dependent and/or BDNF-mediated signaling in cultured cortical neurons (Fig 5A). The level of GAD65 mRNA was significantly reduced with application of AP5, becoming almost the same as with TTX alone (AP5, 0.69 ± 0.05 fold, *p* < .001; TTX, 0.63 ± 0.02 fold, *p* < .001; AP5 vs. TTX, *p* > .05) (Fig 5B). AP5 had an even stronger effect on GAD67 mRNA and protein (S3A, S3B and S3C Fig). Additionally, the expression of both GAD isoforms was not affected by NBQX, a selective blocker of AMPA-Rs (S3A, S3B and S3C Fig). Consistent with the great reduction in GAD67 transcript levels produced by AP5 treatment, immunocytochemical analyses showed that GAD67 immunoreactivity was also significantly reduced in AP5-treated neurons (S3D and S3E Fig), whereas that of vGluT1 was not affected by AP5 treatment (S3D and S3F Fig). These data indicate that spontaneous NMDA-R activity is necessary for basal GAD expression.

Fig 3 demonstrates that BDNF-TrkB signaling cannot be the only mechanism underlying the up-regulated expression of both GAD isoforms under chronically increased activity (Fig 3), suggesting that other Ca^2+^-dependent and BDNF-independent mechanisms contribute. Indeed, inhibition of BDNF signaling by K252a or U0126 did not change the baseline GAD activity (Fig 5B). These data suggest that basal expression of GAD65 is largely maintained by spontaneous NMDA-R activity, a pathway not likely to be mediated by BDNF. In addition to these findings, the reduction in absolute BDNF mRNA levels upon blockade of neuronal activity by TTX and AP5 was significant but very subtle, in contrast to the dramatic increase produced by bicuculline treatment (S2C Fig). This supports the notion that BDNF is not likely to be a major factor for maintenance of basal GAD65 expression. To confirm whether the observed reduction in GAD expression through activity deprivation is mainly due to the silencing of NMDA-R activity by TTX, we further examined the effect of TTX on basal expression of the two GAD isoforms in the presence of AP5 (Fig 5C). The inhibitory effect of chronic TTX on GAD65/67 mRNA expression was completely occluded by AP5 (TTX vs. TTX plus AP5, *p* > .05, non-significant) (Fig 5D), meaning that TTX largely has a significant effect on GAD expression via the reduction of spontaneous NMDA-R activity. Similarly, GAD67 immunoreactivity on the dendrites was dramatically reduced in TTX or AP5-treated neurons, but no additive effect was observed when TTX treatment was combined with AP5 (Fig 5E and 5F). Conversely, vGluT1 immunoreactivity was not affected by any treatments (Fig 5E and 5F). Additionally, we checked that there was no significant difference in the number of neurons between these drug-treated groups (S4 Fig), indicating that alteration in immunoreactivity on dendrites is not due to the reduced number of neurons upon long-term activity deprivation with AP5/TTX treatment. These experiments reveal that spontaneous NMDA-R activity plays a major role in the maintenance of basal expression levels for both GAD isoforms in a BDNF-independent manner.

### Chronic changes in neuronal activity bidirectionally alters enhanced GABA release

**Fig 6:**
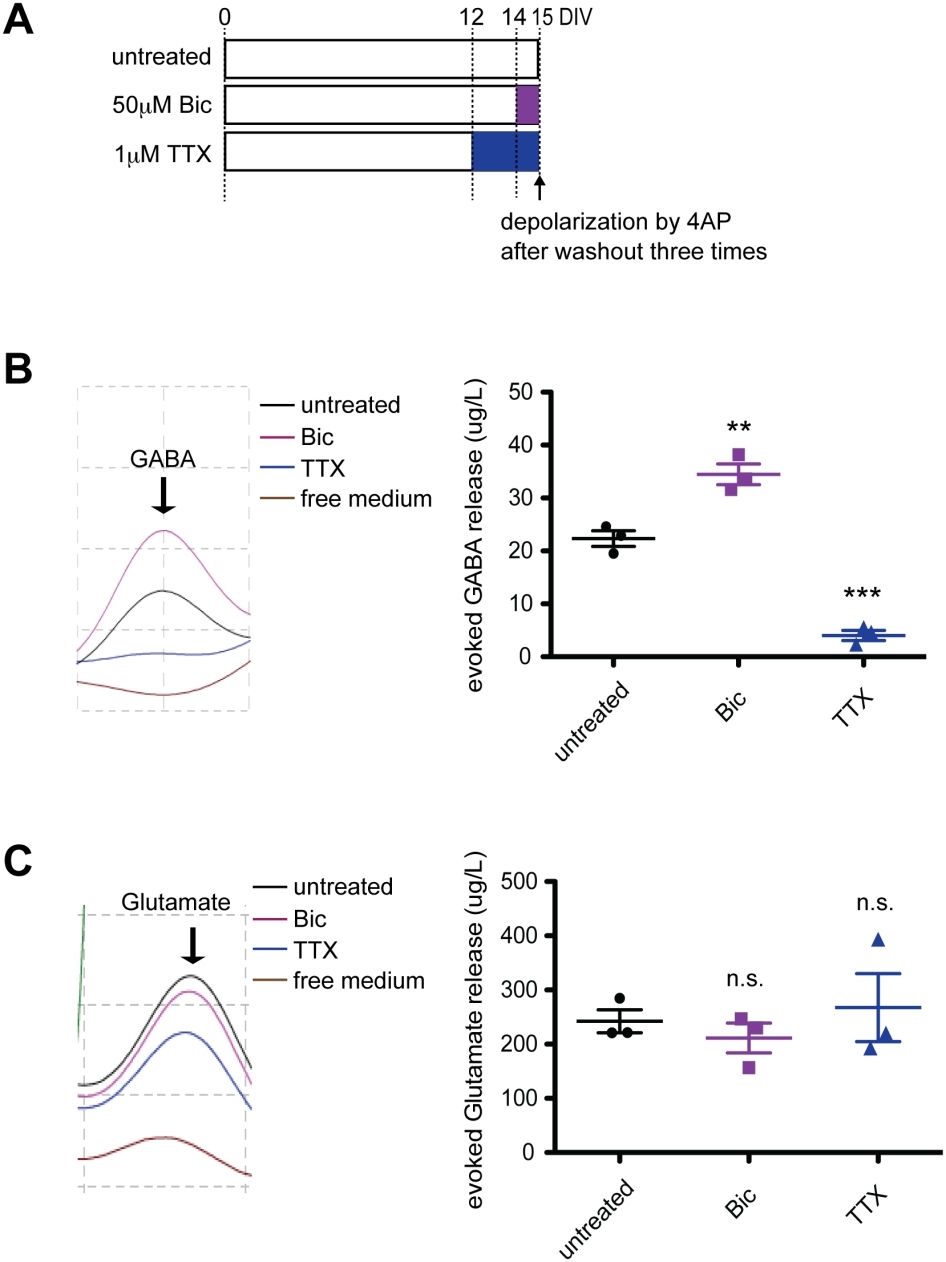
Bidirectional regulation of enhanced GABA release via chronic changes in neuronal activity. (A) Cultured cortical neurons treated with 50 μM bicuculline or 1 μM TTX on DIV14 or on DIV12, respectively, were depolarized with 4-AP on DIV15 to test enhanced neurotransmitter release. (B) Enhanced GABA release following chronic changes in neuronal activity. F(2, 6) = 101.0 (*p* < .0001), one-way ANOVA. (C) Enhanced glutamate release following chronic changes in neuronal activity. F(2, 6) = 0.46 (no significant difference, *p* = 0.7), one-way ANOVA. The right trace in (B) and (C) shows the representative chromatographic peaks of neurotransmitters on HPLC in the representative experiment. Data were analyzed from 3 independent cultures.

Finally, we explored the functional relevance of activity-regulated GAD expression for homeostatic mechanisms. Activity deprivation leads to reductions in both the amplitude and frequency of mIPSCs in a homeostatic manner [4] [23], suggesting that neuronal activity regulates the quantal level of inhibitory synaptic transmission in a homeostatic fashion. To directly assess GAD-related functions at inhibitory presynapses during chronic neuron-activity change, we tested depolarization-induced neurotransmitter release in cultured cortical neurons after treatment with bicuculline or TTX (Fig 6A). GABA or glutamate release from TTX- or bicuculline-treated neurons was enhanced by depolarization with 4-aminopyridine (4-AP), a potassium channel blocker. Depolarization-induced GABA release was significantly enhanced in neurons pre-treated with bicuculline and significantly attenuated in those pre-treated with TTX (Fig 6B), whereas depolarization-induced glutamate release did not show any significant alteration (Fig 6C). Thus, we revealed that the bidirectional form of depolarization-induced GABA release is induced by chronic changes in neuronal activity in a homeostatic fashion. This is in parallel to the dynamic expression of GAD isoforms.

## Discussion

### Activity-regulated expression of GAD isoforms via a BDNF-TrkB signaling-dependent mechanism during chronically increased activity

**Fig 7:**
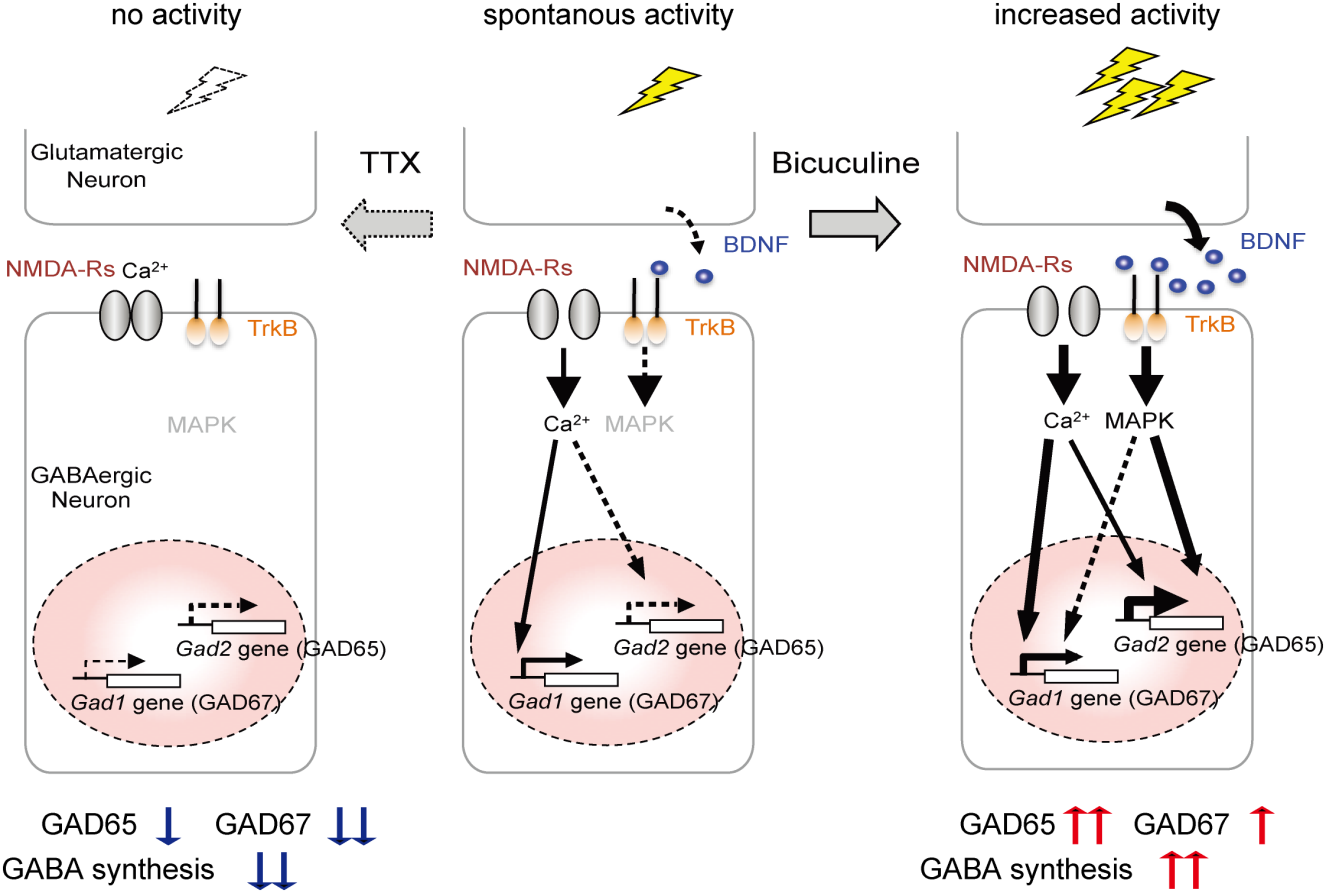
Proposed model of activity-dependent GAD expression through BDNF-dependent and independent pathways triggered by NMDA-R activation. The basal level of GAD expression is largely determined by spontaneous NMDA-R activity on GABAergic neurons. When neuronal activity is persistently increased, expression of both GAD isoforms is up-regulated by increased Ca^2+^ influx into the GABAergic neurons. In parallel, activity-dependent expression of BDNF is also induced by NMDA-R activation through CaMK and MAPK in neighboring excitatory neurons. Activity-regulated BDNF subsequently accelerates GAD expression through TrkB-MAPK signaling in GABAergic neurons. With increased activity, GAD65 is preferentially up regulated by a BDNF-dependent and non-cell autonomous mechanism (right). Conversely, when neuronal activity is silenced, the attenuated Ca^2+^ level in GABAergic neurons leads to a reduction in basal GAD expression. Activity deprivation has a stronger effect on the expression of GAD67 than of GAD65. Overall, we speculate that GABA synthesis may be bidirectionally regulated in a homeostatic fashion by this activity-dependent expression of two GAD isoforms through the interplay of BDNF-dependent and independent pathways triggered by NMDA-R activation.

The present study revealed that chronically increased activity induced by bicuculline leads to the up-regulation of GAD expression (Figs 1 and 2). Consistent with reports that BDNF promotes GAD expression [16] [17], our pharmacological studies involving inhibition of BDNF signaling demonstrated that this up-regulation is BDNF-mediated. Given that GABAergic synapses are regulated by activity in the surrounding neurons [23], we speculate that activity-dependent increases in GADs are facilitated by NMDA-induced BDNF, possibly derived from surrounding excitatory neurons (Fig 7, model, right). BDNF is not likely to be sufficient for inducing an activity-regulated overall change in the expression of GADs (Figs 3 and 4). However, this might not be the case during neural development. Indeed, genetic disruption of activity-regulated BDNF synthesis reduces GAD65 levels and impairs the development of inhibitory synapses [24]. However, all experiments in the present study were performed using mature cultured neurons. Additionally, our pharmacological approaches were unsuited for addressing developmental questions. Moreover, we demonstrated a GAD isoform-dependent responsiveness to BDNF, with GAD65 being more responsive (Fig 2). Therefore, BDNF-dependency could underlie the remarkable difference in up-regulation between GAD65 and GAD67 seen during chronically increased activity. However, sufficiently early in development, GAD67 expression appears to depend also on the BDNF signal, since interneuron-specific genetic disruption of TrkB reduces GAD67 levels at an early postnatal stage but not at a later stage [17]. This suggests that the dependency of GAD67 expression on BDNF signaling is altered during neural differentiation and maturation. One possible mechanism for this is a developmental epigenetic modification such as histone acetylation. Some epigenetic events are activity-dependent and reversible as opposed to inheritable and permanent [25]. Activity-dependent epigenetic events likely implement adaptations to persistent and long-lasting environmental change [25]. A recent study reported differential epigenetic regulation of two GAD genes in cases of persistent pain [26]. Additionally, histone acetylation in the proximal promoter region of an immediate early gene, *Arc*, was found to regulate one of the responses to BDNF stimulation [27]. Therefore, differential sensitivities to BDNF might be due to different epigenetic modifications of the *Gad1* and *Gad2* promoter regions, affecting transcription.

### Activity-regulated expression of GAD isoforms not mediated by BDNF-TrkB signaling

The present study showed that activity-dependent GAD expression triggered by NMDA-R activity is mediated not only through a BDNF-dependent but also through a BDNF-independent mechanism. Indeed, our experiments in Fig 5 demonstrated that TTX-induced down-scaling of GAD65 sufficiently occurs upon inhibition of BDNF-TrkB signaling, suggesting that maintenance of basal GAD expression is largely governed by a BDNF-TrkB independent mechanism. However, we could not completely remove the contribution of BDNF-TrkB signaling. The most likely independent mechanism is cell-autonomous Ca^2+^-dependent regulation via NMDA-R activity expressed by the GABAergic inhibitory neurons (Fig 7, model). Indeed, genetic ablation of the NMDA-R NR1 subunit from interneurons leads to gross reductions in GAD67 and parvalbumin levels [28], strongly suggesting the presence of cell-autonomous regulation of GAD expression by NMDA-Rs in GABAergic neurons. During activity deprivation with TTX or AP5, we observed a significant difference in the extent of reduced expression levels between the two GAD isoforms, GAD67 being dramatically reduced, and GAD65 being significantly but not strongly reduced (Figs 1, 2, and 4). The silencing of isolated hippocampal interneurons by Kir2.1 does not strongly affect GAD65 expression [23]. Beyond a BDNF dependency, the possible presence of additional differences in gene regulation between GAD65 and GAD67 should be explored in future studies.

### Potential implications for activity-dependent gene regulation of GAD isoforms during bidirectional homeostatic scaling in GABAergic neurons

The present study demonstrated that chronic changes in neuronal activity lead to bidirectional alterations in depolarization-enhanced GABA release in a homeostatic fashion. This is consistent with the presence of activity-dependent dynamic regulation of GAD isoform expression. Therefore, the current study proposes the notion that the control of GABA synthesis through activity-dependent GAD expression may be relevant to homeostatic GABA release. Given that long-term exposure to BDNF potentiates depolarization-induced GABA release [16], BDNF-dependent GAD expression may play an important role in up-scaling GABA synthesis (Fig 7, model, right). Conversely, a large part of the TTX-induced down-scaling of GABA synthesis may result from down-regulation of GADs with a BDNF-independent mechanism (Fig 7, model, left). In this case, down-regulation of GAD67 would be more critical for down-scaling GABA synthesis because activity deprivation more severely affected expression of the GAD67 isoform (Figs 1 and 4).

Additionally, previous reports suggest that down-regulation of GAD67 expression during inactivity contributes to TTX-induced homeostatic scaling. For example, *Gad1* knockout was found to occlude TTX-induced homeostatic down-scaling of mIPSCs [29]. These reports also supported the idea that GAD expression is involved in some aspect of bidirectional homeostatic plasticity. Furthermore, given that GAD65 and GAD67 are widely co-expressed in inhibitory neurons, synergism between BDNF-dependent and -independent scaling of GAD expression would enable effective bidirectional homeostatic plasticity of GABAergic presynapses. However, whether activity-dependent expression of GADs is indeed a core mechanism underlying bidirectional forms of homeostatic plasticity remains to be addressed in future studies.

Importantly, since perturbation of the GABA-mediated inhibitory system is implicated in the pathogenesis of not only epilepsy but also several neuropsychiatric disorders, such as autism, schizophrenia, depression, and anxiety, we suggest that transcriptional control of GADs may serve as a potential therapy for the restoration of impaired GABAergic inhibitory functions [30]. Therefore, further elucidation of GAD gene regulation in future studies may provide profound insights into the clinical aspects of these neurological disorders.

## Supporting Information

S1 Fig Activity-dependent expression of GAD65 requires a transcriptional event. (A) Cultured cortical neurons were treated with bicuculline (50 μM), a transcriptional inhibitor, actinomycin D (1 μg/ml), or bicuculline plus actinomycin D on DIV15 and harvested 12 hours after treatment. (B) Relative GAD65 mRNA expression levels in cultured cortical neurons treated as shown in (A). F(3, 8) = 5.841 (*p* < .02), one-way ANOVA. (*n* = 3 per each treatment).
(TIF)

S2 Fig Activity-dependent expression of BDNF mRNA and protein. Relative BDNF mRNA (A) and protein (B) expression levels after treatment with bicuculline (50 μM) in the presence of K252a (100 nM), U0126 (10 μM), AP5 (200 μM), or KN93 (5 μM). Note that K252a does not disrupt activity-dependent BDNF expression. mRNA: F(5, 62) = 60.49 (*p* < .0001); protein: F(3, 8) = 25.47 (*p* = .0002), one-way ANOVA. (n = 4–11 per each treatment). (C) Relative BDNF mRNA expression levels after treatment with bicuculline (50 μM), TTX (1 μM), K252a (100 nM), U0126 (10 μM), or AP5 (200 μM) in cultured cortical neurons after 3 days in the culture. The neurons are harvested on DIV15. Note that the absolute level of basal BDNF expression is significant but is not altered by activity deprivation compared with activity increase. F(5, 47) = 49.93 (*p* < .0001), one-way ANOVA. (*n* = 4–6 per each treatment).
(TIF)

S3 Fig Activity deprivation via the selective NMDA-R inhibitor (AP5) reduces basal GAD expression. (A) Cultured cortical neurons are treated with AP5 (200 μM) or NBQX (20μM) on DIV12 for the last 3 days in the culture and are harvested on DIV15. (B) Relative mRNA expression levels of presynaptic molecules in cultured cortical neurons treated with AP5 or NBQX as shown in (C). GAD65: F(2, 12) = 23.31 (*p* < .0001); GAD67: F(2, 12) = 78.42 (*p* < .0001); vGluT1: F(2, 12) = 1.092 (no significant difference, *p* = .36), vGAT: F(2, 10) = 13.03 (*p* = .0016) one-way ANOVA. (n = 6, AP5; n = 3, NBQX). (C) Relative protein levels of GAD67, vGluT1, NR1, and GluR2/3 measured by immunoblotting. Inactivity selectively reduces GAD67 protein expression. GAD67: F(2, 29) = 61.47 (*p* < .0001); NR1: F(2, 8) = 3.032 (no significant difference, *p* = .1), one-way ANOVA. (*n* = 7–9 per each treatment). (D) Representative immunocytochemistry images of endogenous GAD67 protein in AP5- or NBQX-treated neurons. Cultured cortical neurons are triple-stained with anti-GAD67, anti-vGluT1, and MAP2 antibodies. (E, F) Intensities of GAD67 and vGluT1 immunoreactivity measured in MAP2-positive dendritic areas. Twelve confocal images in each condition are analyzed from 3 independent experiments. GAD67: F(2, 33) = 7.424 (*p* < .002); vGluT1: F(2, 33) = 0.2446 (no significant difference, *p* = .78), one-way ANOVA. Scale bar = 50 μm.
(TIF)

S4 Fig Long-term activity deprivation via TTX or the selective NMDA-R inhibitor (AP5) does not severely influence neuron survival in matured cortical neurons. (A) Representative images of co-immunocytostaining with antibodies against the neuronal cell markers anti-MAP2 and anti-NeuN in TTX- and TTX plus AP5-treated cortical cultures. Co-immunostaining was combined with nuclear staining using DAPI. The sister cultures were used in Fig 5 in these experiments. Scale bar = 100 μm. (B) Quantification of neuronal cell number in TTX- and TTX plus AP5-treated cortical cultures shown in (A). Low magnification images were captured on an Axio Plan2 (ZEISS). The number of MAP2^+^NeuN^+^ neuronal cells was counted and averaged on 10 separate areas (0.2 mm^2^) randomly chosen on the images for each group per experiment. The cell densities (×10^2^/mm^2^) were estimated in 2 independent experiments. F(2, 27) = 14.40 (no significant difference, *p* = .093) one-way ANOVA.
(TIF)
