# Supplementary figures and images for: Activity-Dependent Bidirectional Regulation of GAD Expression in a Homeostatic Fashion Is Mediated by BDNF-Dependent and Independent Pathways
Source: PLoS One. 2015 Aug 4;10(8):e0134296. doi: 10.1371/journal.pone.0134296 (PMC4524701; doi:10.1371/journal.pone.0134296)

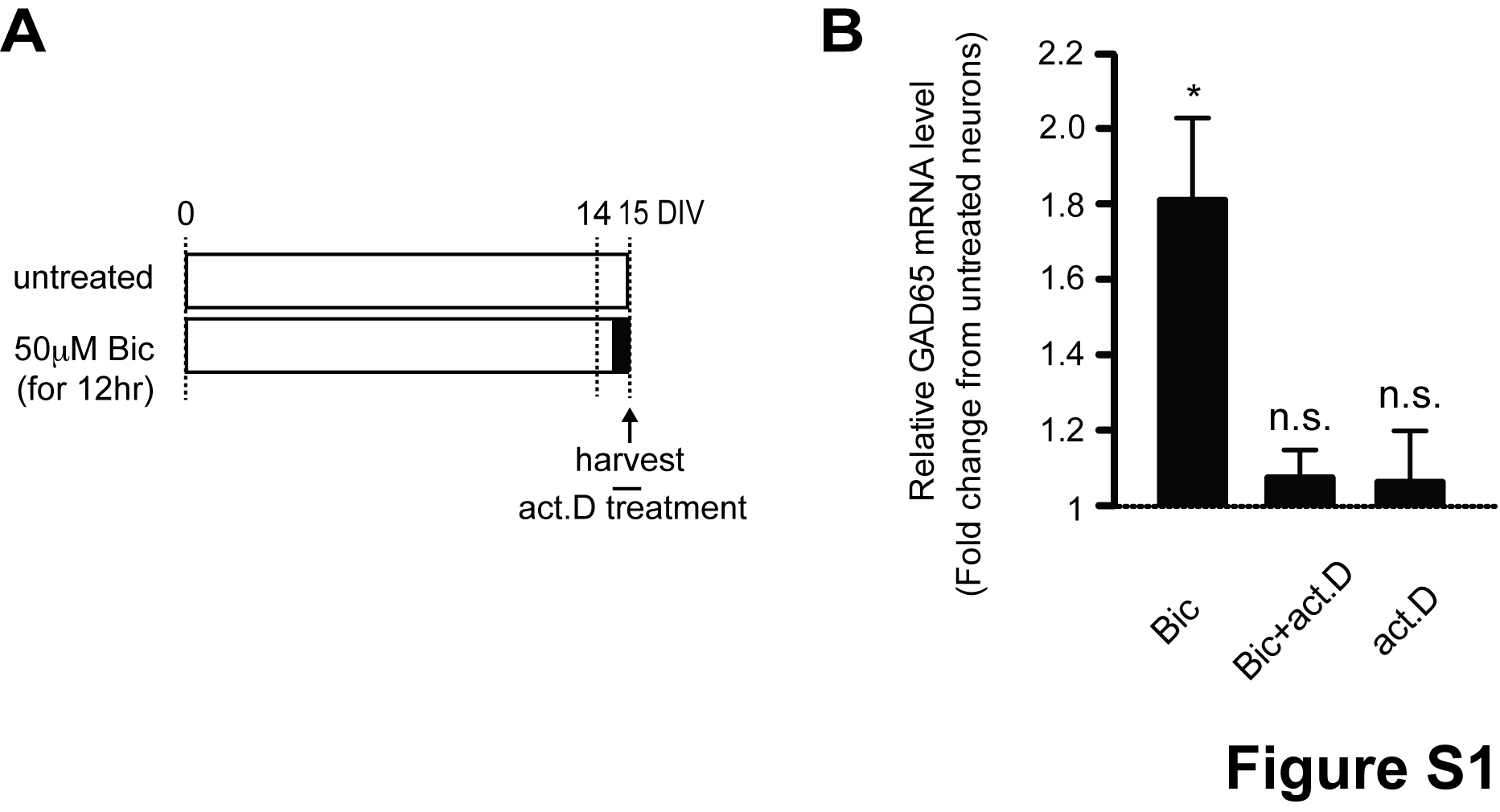

Supplement: S1 Fig — (A) Cultured cortical neurons were treated with bicuculline (50 μM), a transcriptional inhibitor, actinomycin D (1 μg/ml), or bicuculline plus actinomycin D on DIV15 and harvested 12 hours after treatment. (B) Relative GAD65 mRNA expression levels in cultured cortical neurons treated as shown in (A). F(3, 8) = 5.841 (p < .02), one-way ANOVA. (n = 3 per each treatment). (TIF) [file pone.0134296.s001.tif]

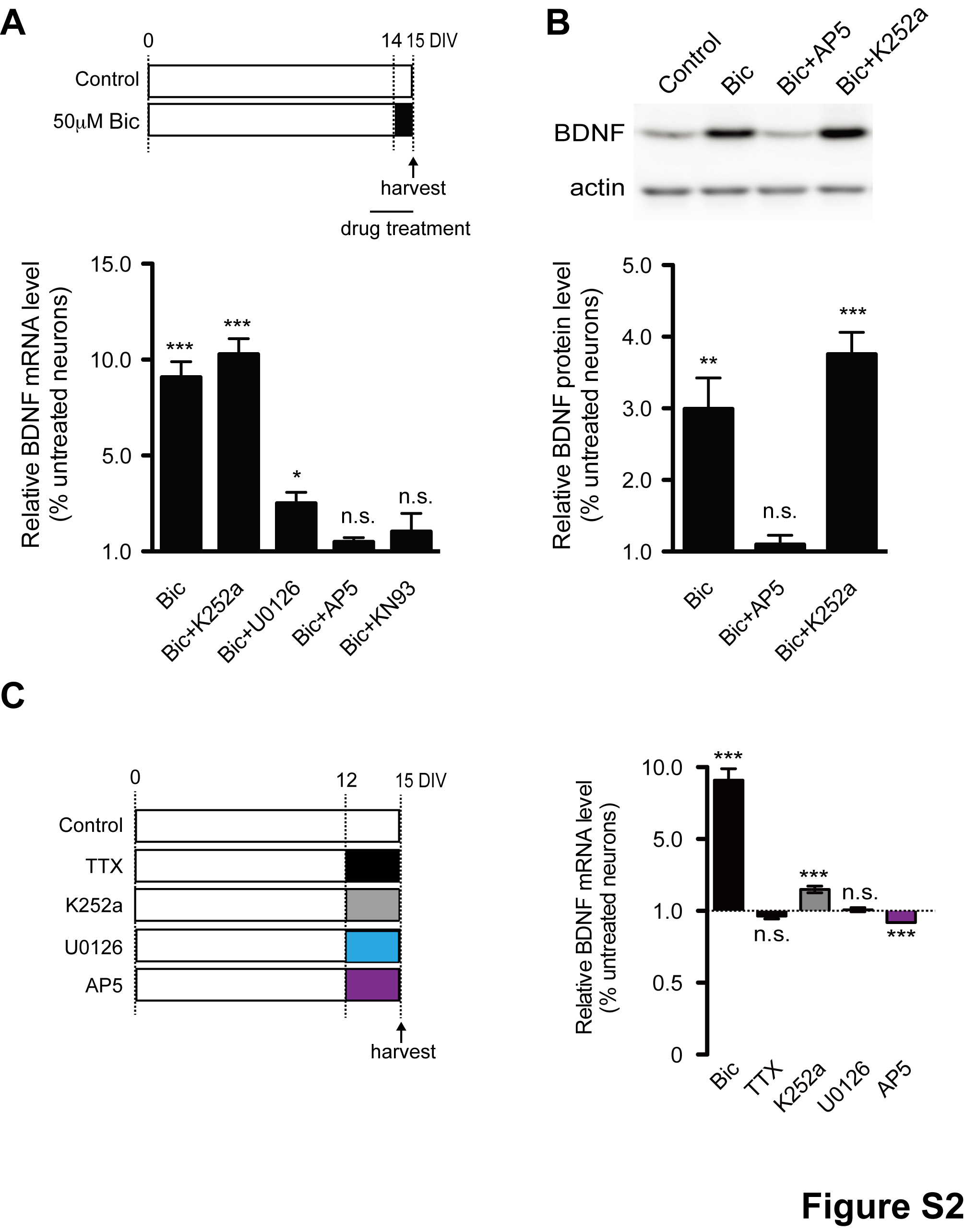

Supplement: S2 Fig — Relative BDNF mRNA (A) and protein (B) expression levels after treatment with bicuculline (50 μM) in the presence of K252a (100 nM), U0126 (10 μM), AP5 (200 μM), or KN93 (5 μM). Note that K252a does not disrupt activity-dependent BDNF expression. mRNA: F(5, 62) = 60.49 (p < .0001); protein: F(3, 8) = 25.47 (p = .0002), one-way ANOVA. (n = 4–11 per each treatment). (C) Relative BDNF mRNA expression levels after treatment with bicuculline (50 μM), TTX (1 μM), K252a (100 nM), U0126 (10 μM), or AP5 (200 μM) in cultured cortical neurons after 3 days in the culture. The neurons are harvested on DIV15. Note that the absolute level of basal BDNF expression is significant but is not altered by activity deprivation compared with activity increase. F(5, 47) = 49.93 (p < .0001), one-way ANOVA. (n = 4–6 per each treatment). (TIF) [file pone.0134296.s002.tif]

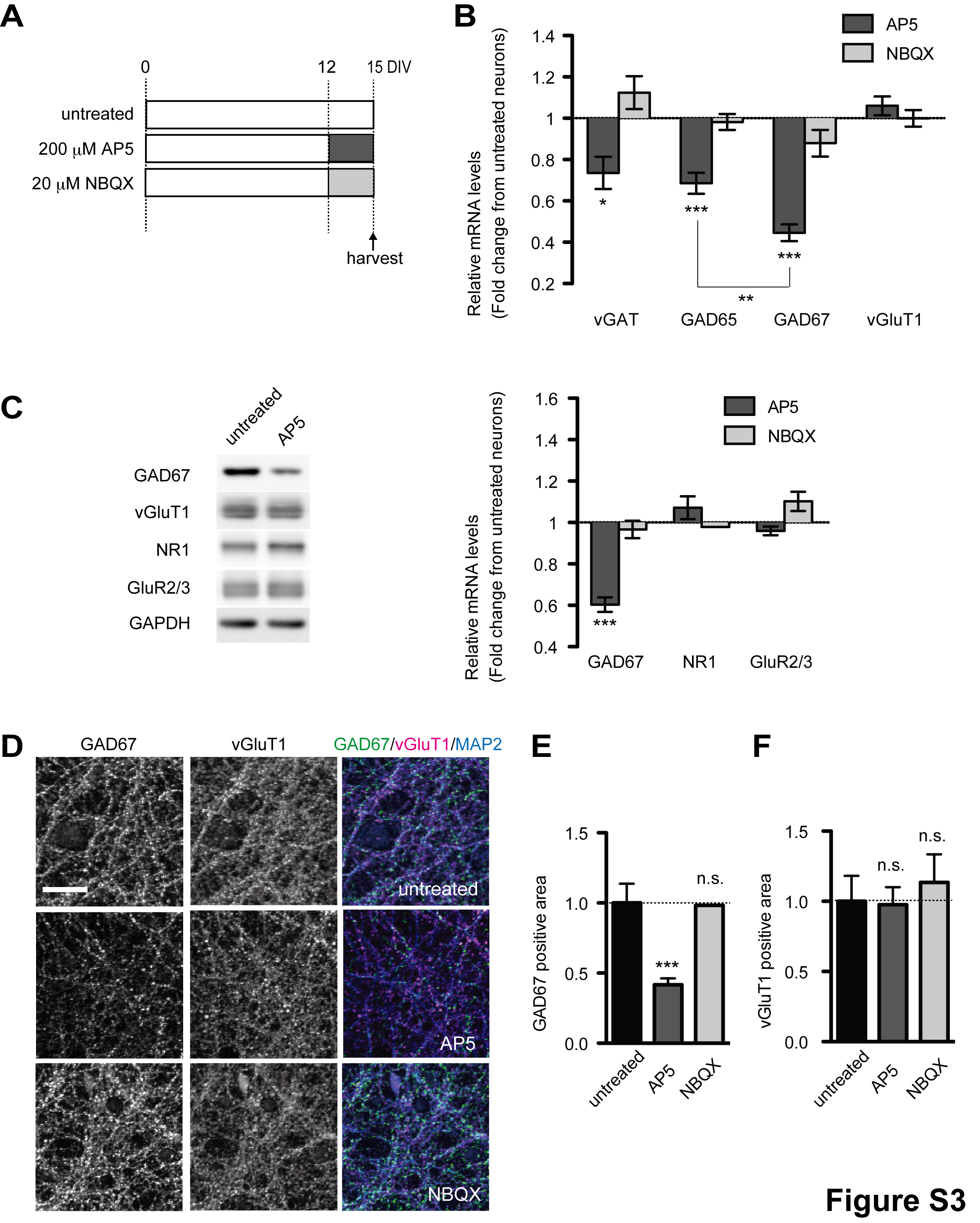

Supplement: S3 Fig — (A) Cultured cortical neurons are treated with AP5 (200 μM) or NBQX (20μM) on DIV12 for the last 3 days in the culture and are harvested on DIV15. (B) Relative mRNA expression levels of presynaptic molecules in cultured cortical neurons treated with AP5 or NBQX as shown in (C). GAD65: F(2, 12) = 23.31 (p < .0001); GAD67: F(2, 12) = 78.42 (p < .0001); vGluT1: F(2, 12) = 1.092 (no significant difference, p = .36), vGAT: F(2, 10) = 13.03 (p = .0016) one-way ANOVA. (n = 6, AP5; n = 3, NBQX). (C) Relative protein levels of GAD67, vGluT1, NR1, and GluR2/3 measured by immunoblotting. Inactivity selectively reduces GAD67 protein expression. GAD67: F(2, 29) = 61.47 (p < .0001); NR1: F(2, 8) = 3.032 (no significant difference, p = .1), one-way ANOVA. (n = 7–9 per each treatment). (D) Representative immunocytochemistry images of endogenous GAD67 protein in AP5- or NBQX-treated neurons. Cultured cortical neurons are triple-stained with anti-GAD67, anti-vGluT1, and MAP2 antibodies. (E, F) Intensities of GAD67 and vGluT1 immunoreactivity measured in MAP2-positive dendritic areas. Twelve confocal images in each condition are analyzed from 3 independent experiments. GAD67: F(2, 33) = 7.424 (p < .002); vGluT1: F(2, 33) = 0.2446 (no significant difference, p = .78), one-way ANOVA. Scale bar = 50 μm. (TIF) [file pone.0134296.s003.tif]

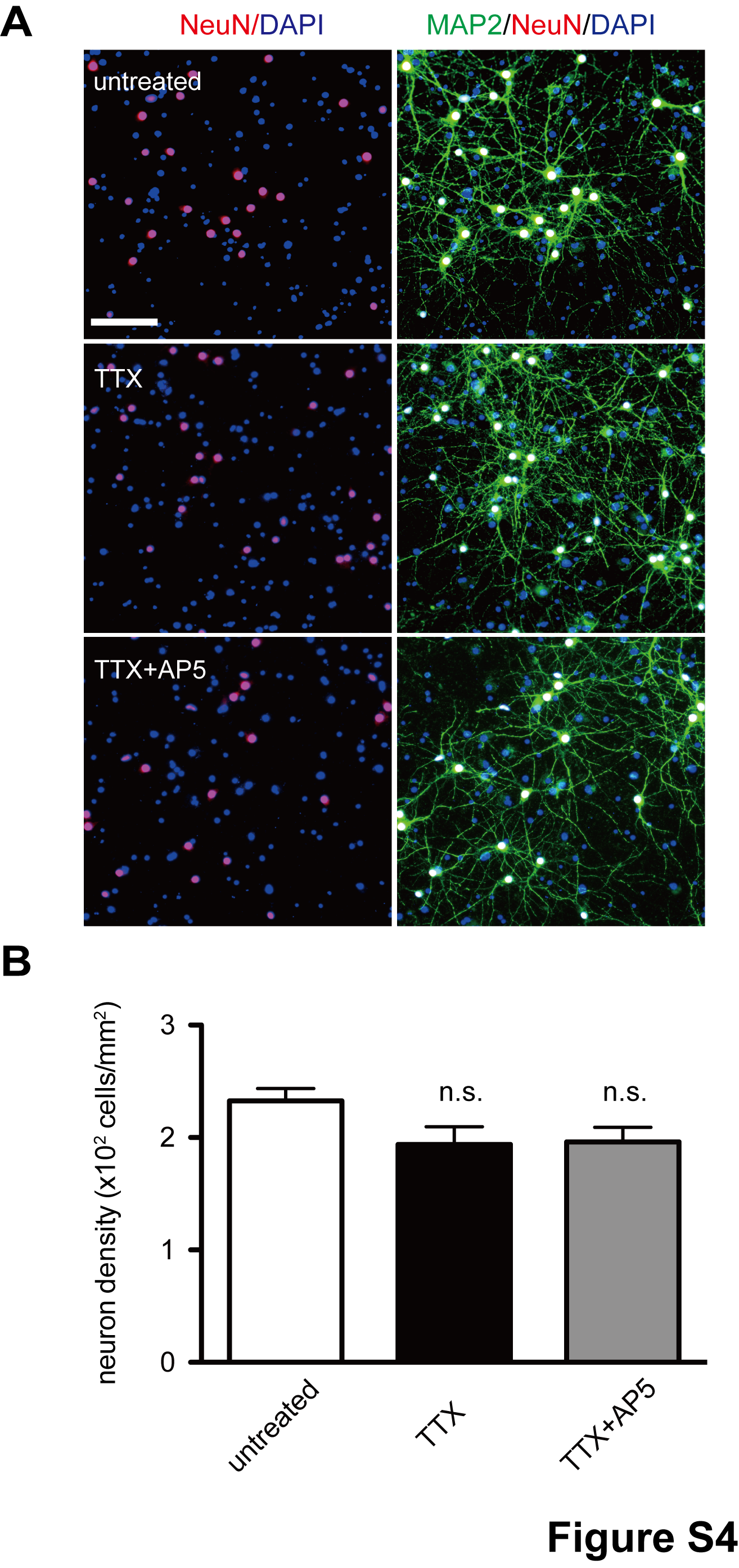

Supplement: S4 Fig — (A) Representative images of co-immunocytostaining with antibodies against the neuronal cell markers anti-MAP2 and anti-NeuN in TTX- and TTX plus AP5-treated cortical cultures. Co-immunostaining was combined with nuclear staining using DAPI. The sister cultures were used in Fig 5 in these experiments. Scale bar = 100 μm. (B) Quantification of neuronal cell number in TTX- and TTX plus AP5-treated cortical cultures shown in (A). Low magnification images were captured on an Axio Plan2 (ZEISS). The number of MAP2+NeuN+ neuronal cells was counted and averaged on 10 separate areas (0.2 mm2) randomly chosen on the images for each group per experiment. The cell densities (×102/mm2) were estimated in 2 independent experiments. F(2, 27) = 14.40 (no significant difference, p = .093) one-way ANOVA. (TIF) [file pone.0134296.s004.tif]
